# Woman-Sensitive One Health Perspective in Four Tribes of Indigenous People From Latin America: Arhuaco, Wayuú, Nahua, and Kamëntsá

**DOI:** 10.3389/fpubh.2022.774713

**Published:** 2022-03-07

**Authors:** Natalia Margarita Cediel-Becerra, Silvana Prieto-Quintero, Angie Daniela Mendez Garzon, Mindhiva Villafañe-Izquierdo, Clara Viviana Rúa-Bustamante, Nathaly Jimenez, Jairo Hernández-Niño, Julie Garnier

**Affiliations:** ^1^Facultad de Ciencias Agropecuarias, Universidad de La Salle, Bogotá, Colombia; ^2^Hospital José Antonio Socarras, Sala de Emergencias, Manaure, Sucre, Colombia; ^3^Centro de Investigación Motilonia, Km 5 vía Becerril, Agustín Codazzi, Cesar, Corporación Colombiana de Investigación Agropecuaria-Agrosavia, Mosquera, Colombia; ^4^Universidad del Rosario, Bogotá, Colombia; ^5^Department of Biology, Universidad Internacional del Trópico Americano, Unitrópico, Yopal, Colombia; ^6^Odyssey Conservation Trust, Bakewell Derbyshire, United Kingdom

**Keywords:** gender, ethnic, ecohealth approach, transdisciplinary collaboration, Latin America, sustainable development goal (SDG)

## Introduction

There is now a growing concern for the health and well-being of estimated 45 million indigenous people living in the region of America due to health inequities, poor environmental justice, poor social policies and programs, unfair economic arrangements, power relationships, and impacts of colonization, discrimination, and marginalization (1–3). According to the Health of Indigenous Peoples Initiative, five key principles are central to indigenous people's health: the need for a holistic approach to health, the right to self-determination of indigenous peoples, the right to systematic participation, respect for and revitalization of indigenous cultures, and reciprocity of relations (2, 4). Although they account for only around five percent of the world's population, they effectively manage an estimated 20–25 percent of the Earth's land surface. Indigenous people also own, occupy, or manage land, holds 80% of the planet's biodiversity and intersects with about 40% of all terrestrial protected areas and ecologically intact landscape. Biodiversity conservation and cultural diversity, therefore, cannot be dissociated from the stewardship of indigenous people over their natural resources, but this fundamental aspect of One Health has been neglected previously (5, 6). Indigenous women have been recognized to play a key role in sustainable development, biodiversity conservation, peace building, and food security despite multiple socioeconomic gaps they face (6–10). There is little knowledge of their relative influence on management of the Human-Animal-Environment interface in a One Health context. The objective of our article is to explore gaps and opportunities from a woman-sensitive One Health perspective in four tribes of indigenous people located in Colombia and Mexico, while recognizing the added value of integrating nonacademic knowledge into the One Health practice and draw attention to the need for including and considering indigenous women's voice, wisdom, and practices. The four cases describe the relationship of women with nature through their understanding of their natural environment, traditional knowledge, wisdom, practices, and current challenges. Semi-structured interviews were conducted with Arhuaco and Kamëntsá people while for Wayuú and Nahua people, secondary information was used from published literature from co-authors of this manuscript and others related. Our questions explored the following issues:

- What traditions, knowledge, and practices do indigenous women consider as being most relevant for preserving biodiversity and ensuring well-being and welfare?
- What is the perceived impact of climate change on their subsistence?
- What are women's main constraints in their communities in a One Health context?

## Traditions of Knowledge and Practices Most Relevant for Preserving Biodiversity, Well-Being, and Welfare

Mindhivä Villafañe Izquierdo, one of the co-authors of this manuscript, belongs to the Arhuaco people. Life in her community is based on the *Law of Origin*, the traditional science of wisdom and indigenous ancestral knowledge for management of the material and spiritual world (11) and on the interconnectedness of all forms of life, as well as their intrinsic relationship to their territory and belonging to one another.

“*.… In Arhuaco culture, the women are recognized as sagas or spiritual guides, midwives, masseuses, and have a role in preparing plants to treat disease. Women are zaku or aty which means mothers of all existences...The mother is the one who concretizes a dream, a vision, an invisible desire, while men are like air or heaven, the non-concrete seed*.

(Mindhivä Villafañe Izquierdo, Arhuaco woman, medical doctor, October 2021)

Likewise, Hernandez-Niño (co-author) visited and worked with the Nahua community of Los Reyes (Acaxochitlán, Hidalgo, México), where a women's group called “Nanacateras” has maintained a tradition of collecting wild edible mushrooms. The objective of his research was to investigate drivers of biodiversity loss, especially fungi, in this region. He identified that women were in charge of selection, collection, cooking, and trading of wild fungi, and that these traditions were passed down to the next generation through women. Modern day Nanacateras were found to be proud of their role in maintaining these traditions, but they also expressed concern for the damage caused by modern society to their natural resources. The forest they respected and protected with great care was threatened by legal and illegal logging, leading to loss of biodiversity, in particular fungal diversity and abundance (12).

Furthermore, in an interview conducted by co-author Jimenez to a Kamëntsá woman leading the Association of Indigenous Women “*La Chagra de la Vida*” (ASOMI) (13, 14), a grassroots organization located in the department of Putumayo, said that the Kamentsa people spend a large amount of time around their chagras (spaces used by indigenous people to cultivate). There are currently around 709 species of medicinal, food, artisanal, and timber plants being cultivated. ASOMI women “*understand that development beyond being sustainable must be healthy. Healthy food is medicine in its chagras* (15)”. An ASOMI woman explained:

“*The food sovereignty of our peoples is at risk. Native seeds have been lost and transgenic seeds are preferred, driven by the implementation of government projects... our territories are affected by the urban expansion of municipalities that do not respect the use and management of the territory defined by the Peoples: sacred sites, water, the forest, etc... And almost everything becomes garbage that ends up in the bosom of Mother Earth, in the streams, rivers, and finally into the sea* ”

(Kamëntsá Indigenous Woman, Putumayo, ASOMI, September, 2021).

Regarding climate change, Villafañe explained:

“*In our town we have noticed climate change through the decrease in snow in the snow-capped mountains, changes in all places that affect endemic species since those from a cold or temperate climate have had to migrate or others die. There are also visible changes in crops, which due to the need of a certain temperature that has changed, don't grow anymore, affecting families dependent on these crops”*.

Correspondingly, a Kamëntsá woman from Putumayo described how climate change has increased challenges in her community:

“*Based on all these great challenges that we are experiencing in the face of climate change, we no longer have a calendar that ancestrally handled winter and summer times, and we knew about planting times and harvests. Unfortunately, climate change has weakened that ancestral calendar, but we are optimistic and hopeful that if we return to our own sensitivity, reconnect with mother earth, through our seeds we will be able to guarantee a dignified life with fundamental rights as civil society, as indigenous peoples, as Afros and peasants”*.

(Kamëntsá Indigenous Woman, Putumayo, ASOMI, September, 2021)

## Women's Main Challenges in a One Health Context

Wayuú indigenous people (Guajira, Northern Colombia) are described as a matrilineal social organization rooted in their cosmogony (16). As a result, women remain in their maternal territory and take care of their family, while men move throughout the territory, visiting and impregnating their women. Additionally, Wayuú women are in charge of collecting water for their families and livestock, which represents one of their most important daily tasks as it can involve walking over great distances, even if donkeys are sometimes used to help (16). Life is not easy in their community; Wayu' people actually face a humanitarian crisis, as they have the highest children malnutrition rate in Colombia (17). Aviles (18) reported that approximately 5,000 children of the Wayúu tribe died between 2007 and 2017 because of their lack of access to clean water, lack of sufficient food and access to health services, and high poverty rate together with humanitarian crisis of migration from Venezuela, and local government corruption. A severe drought was also a proximate factor to this massive loss of life, but the drought concealed a larger historical, political, and economic context that was fundamental to this health crisis (18).

On the other hand, traditionally, Nanacateras women (from Hidalgo, Mexico) use natural fiber baskets to collect fungi deep into the forest, but over the last forty years, they have been using plastic buckets because of their low cost, durability and multiple utilities, causing loss of traditional weaving knowledge. It was found that the new practice of collecting fungi in plastic baskets had also contributed to the significant depletion in fungi populations through reduced spores dissemination with plastic containers compared with natural fibers baskets. This practice also led to the need for women to walk deeper in the forest to find wild edible fungi. This also highlighted the loss of biocultural heritage faced by the Nahua community through their interaction with modern society and its consumerist and wasteful lifestyles, driving new generations away from their cosmovision, traditions, and understanding of and respect for mother nature (12).

## Discussion

**Figure 1:**
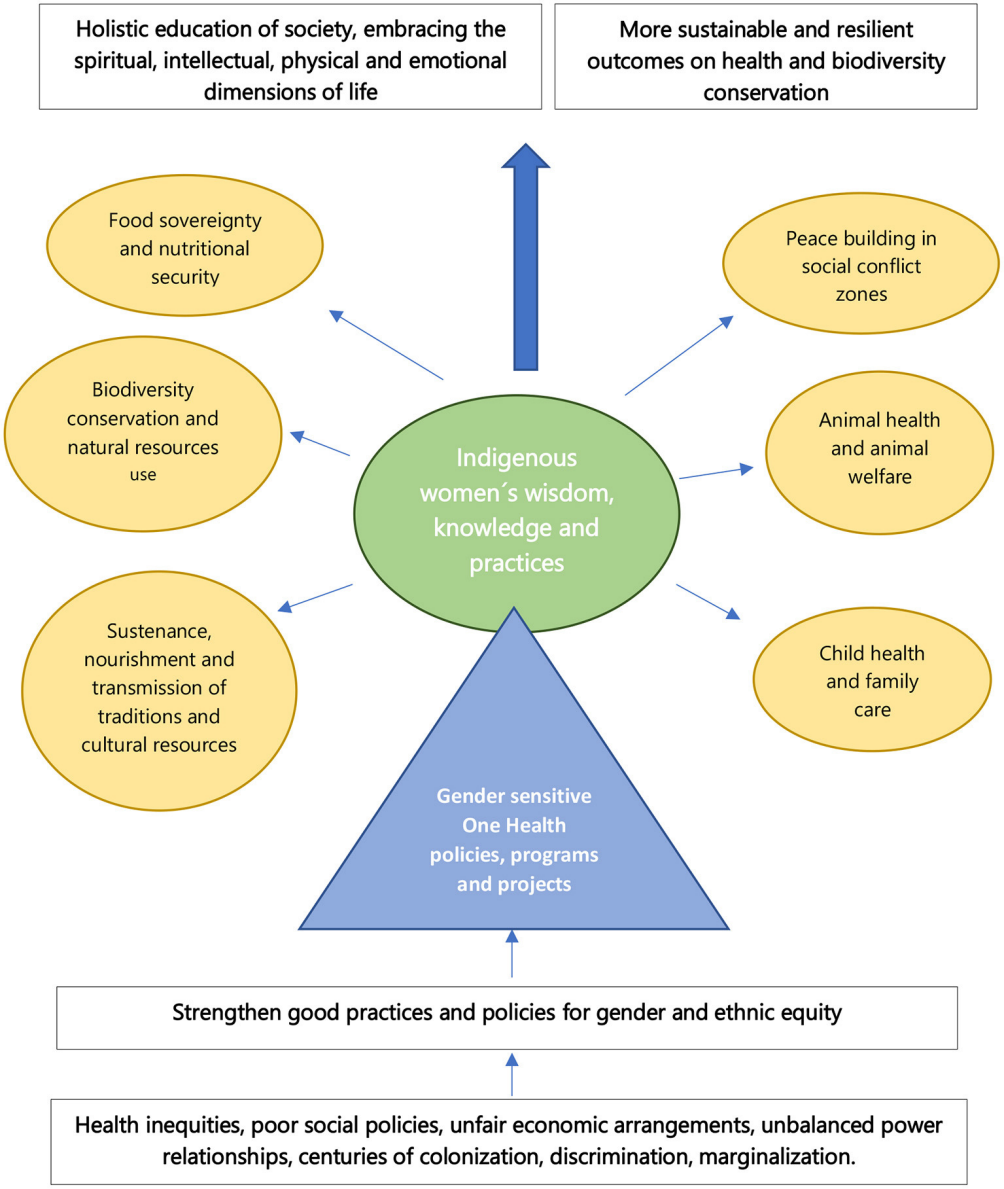
Analytical framework for woman-sensitive One Health perspective in indigenous people.

The One Health approach is still evolving as a science and an evidence-based discipline, with limited published literature demonstrating its added value in real-world settings such as indigenous territories, in unison with scarce evidence of the impact of gender equality on One Health policies, plans, or projects. As systems can be tangible (e.g., humans, animals, forests, and lakes) or intangible (e.g., cultural behaviors, values, norms, and language expressions) and are linked by interactions (19), One Health systems at different levels could learn and benefit from inclusion of indigenous women's worldviews, perspectives, and wisdom. Their concept of health and survival is both collective and individual inter-generational continua encompassing a holistic perspective, which incorporates four dimensions of life: spiritual, intellectual, physical, and emotional. For them, health is viewed as the harmonious coexistence of human beings with nature, with themselves, and with others in pursuit of well-being (2). Actually, the Convention on Biological Diversity pointed out the importance of creating mechanisms to ensure that all relevant stakeholders, including indigenous and local communities, can be involved effectively in the design, implementation, and review of One Health (3). Our four examples illustrate the vital role of indigenous women in preserving their natural and cultural resources, managing agrobiodiversity, and household food and nutritional security through management of small-scale agriculture (20). Various United Nations human rights treaty organizations have expressed concern about their higher child and maternal morbidity and mortality rates, unwanted pregnancy, and sexual abuse derived from structural violence; incidence of chronic diseases caused by environmental pollution and extractive industries; mental health problems primarily affecting youth; and lack of access to culturally sensitive health services (2, 18). Structural inequities like poorer access to land, agricultural inputs, credit, education, extension, and other services compared to men, as well as gender-determined roles and responsibilities in collecting water, firewood, and other cooking fuels, and unpaid care roles reinforce gender inequities (10, 21, 22). Likewise, according to the CBD (3), impacts of environmental degradation and biodiversity loss on health outcomes are most significant among vulnerable populations, specially those most reliant on natural resources and less covered by health coverage such as indigenous women. Despite indigenous peoples' resilience to being threatened by their vulnerability to social, health, and climate crises, they have often been able to adapt to these changes by continuous practice of traditional knowledge passed between generations in their long-established language (5, 23). We observed how traditional knowledge is key to biodiversity and nature conservation along the four narratives presented. Many indigenous women argue that land grabbing, deforestation, extractivism, and privatization of natural resources have worsened their position in recent decades (24), indicating that the lack of gender equity in society mirrors the lack of biodiversity conservation and natural resource protection. Despite indigenous women's unique contribution to maintaining safe environments, achieving food security, and enhancing spiritual well-being of society, it is considered that their traditional knowledge is not valued properly and that in some cases, it has been made invisible (5). In conclusion, we consider that the presence of indigenous women has guaranteed the permanence of their values and traditions; they are protecting traditional medical knowledge and practices, promoting sustainable use of plants and animals, and securing human health, nutrition, and well-being. Without them, the traditions of knowledge they pass down would have disappeared by now, and we would be at a loss of valuable information for the survival of our planet and species. Finally, the definition of One Health developed by the One Health High Level Expert Panel (OHHELP), stated that inclusion and engagement of communities and marginalized voices are key underlying principles for One Health implementation (25), and that the achievement of Sustainable Development Goal number 5 (gender equality) is not only a fundamental human right but a necessary ground for a peaceful, healthy, and sustainable world. See Figure 1 for the description of the analytical framework for woman-sensitive One Health perspective in indigenous people.

## Author Contributions

NMC-B, SP-Q, ADMG, MV-I, CR-B, NJ, JH-N, and JG: writing of the article with equal contribution on the four case studies and article editing. NMC-B and JG: senior author supervising the writing and editing, conceptualization, and design of the manuscript. SP-Q and ADMG: translation of the manuscript. All authors reviewed the final version of the manuscript. All authors contributed to the article and approved the submitted version.

## Funding

This work was supported by Una Salud en Iberoamérica y el Caribe Frente a Cambio Climático y Perdida de Biodiversidad (USCC).

## Conflict of Interest

JG was employed by Odyssey Conservation Trust. The remaining authors declare that the research was conducted in the absence of any commercial or financial relationships that could be construed as a potential conflict of interest. The reviewer BM-F declared a past collaboration with one of the authors NMC-B to the handling editor.

## Publisher's Note

All claims expressed in this article are solely those of the authors and do not necessarily represent those of their affiliated organizations, or those of the publisher, the editors and the reviewers. Any product that may be evaluated in this article, or claim that may be made by its manufacturer, is not guaranteed or endorsed by the publisher.
